# Clinical study on gefitinib combined with γ-ray stereotactic body radiation therapy as the first-line treatment regimen for senile patients with adenocarcinoma of the lung (final results of JLY20080085)

**DOI:** 10.3892/mco.2013.135

**Published:** 2013-05-27

**Authors:** DEJIAN PAN, BIAO WANG, XIJIAN ZHOU, DONGLIN WANG

**Affiliations:** 1 Department of Oncology, 101st Hospital of PLA, Clinical Medical College of Jiangsu University, Wuxi, Jiangsu 214044;; 2 Department of Oncology, Tongji Hospital Affiliated to Tongji University, Shanghai 200065, P.R. China

**Keywords:** gefitinib, γ-ray stereotactic body radiation therapy, adenocarcinoma of lung, senile, first-line regimen

## Abstract

Lung cancer is one of the most commonly encountered human malignancies. Due to the increase in life expectancy as well as the incidence of lung cancer, the incidence of senile lung cancer has increased significantly. We conducted a study to evaluate the efficacy and safety of gefitinib combined with γ-ray stereotactic body radiation therapy (SBRT) as the first-line treatment regimen for senile patients with adenocarcinoma of the lung. A total of 122 senile patients with adenocarcinoma of the lung were divided into 3 groups according to the treatment method. Group A included 35 patients treated with gefitinib combined with γ-ray SBRT, group B included 45 patients treated with γ-ray SBRT alone and group C included 42 patients treated with gefitinib alone. The patients received 250 mg of gefitinib per day, from the first day of the treatment until disease progression or discontinuation due to other causes. The patients were treated with γ-ray SBRT, initiated on the second day. The dose curve for this case group was 50–80%. The encircled dose was 4.0–6.5 Gy per fraction and the range of the total radiation dose was 36–48 Gy. The total number of treatments was 8–12, at a frequency of 5 times per week. All 122 patients were assessed by contrast-enhanced double helical computed tomography (CT) at 2 months. The tumor response rate (RR) of group A was 68.6% (24/35), the disease control rate (DCR) was 88.6% (31/35), the median overall survival (OS) was 15.5 months (range, 3–52 months) and the progression-free survival (PFS) was 7.8 months. The 1-year OS rate was 40.0% (14/35). The main side effects included skin rash and diarrhea. The RR of group B was 51.1% (23/45), the DCR was 71.1% (32/45), the OS was 9.6 months (range, 3–22 months) and the PFS was 5.3 months. The 1-year OS rate was 15.6% (7/45). The RR of group C was 40.5% (17/42), the DCR was 61.9% (26/42), the OS was 10.3 months (range, 3–26 months) and the PFS was 5.1 months. The 1-year OS rate was 35.7% (15/42). The main side effects included skin rash and diarrhea. The short-term therapeutic effect (RR) in group A was higher compared to that in group C (P=0.014, χ^2^=6.053); however, there was no significant difference compared to group B (P=0.116, χ^2^=2.477). The PFS of group A was higher compared with that of groups B and C (7.8 vs. 5.9, P=0.018 and 7.8 vs. 5.1, P=0.013, respectively). The OS of group A was higher compared with that of groups B and C (15.5 vs. 9.6, P=0.002 and 15.5 vs. 10.3, P=0.017, respectively). No significant differences were observed in PFS and OS between groups B and C. In conclusion, gefitinib combined with γ-ray SBRT appears to be feasible and effective as the first-line treatment in senile patients with adenocarcinoma of the lungs.

## Introduction

Lung cancer is one of the most commonly encountered human malignancies. Due to the increase in life expectancy and the incidence of lung cancer, the incidence of senile lung cancer has increased significantly and reaches a peak between the ages of 70 and 74 years. Adenocarcinoma accounts for ∼50% of lung cancer cases in senile patients. Therefore, the combined modality treatment of lung adenocarcinoma is important for senile patients. Radiotherapy and biological targeted therapy have become important therapeutic methods for senile patients with adenocarcinoma of the lung, due to organ miopragia and decreased cell damage repair ability exhibited by senile patients. Gefitinib (ZD1839, Iressa) is a selective epidermal growth factor receptor (EGFR) tyrosine kinase inhibitor, exhibiting satisfactory effects and low adverse reaction rates in Asian non-small cell lung cancer patients (NSCLC) (1). Gefitinib has also been used in the treatment of elderly or poor performance status patients with advanced NSCLC (2). γ-ray stereotactic radiotherapy (SBRT) exerts a satisfactory short-term therapeutic effect in senile NSCLC patients. Furthermore, EGFR inhibitors have been proven to be efficient radiosensitizers (3,4). In order to evaluate the efficacy and safety of gefitinib combined with γ-ray SBRT as the first-line treatment for senile patients with adenocarcinoma of the lung, we enrolled 122 senile lung adenocarcinoma patients between July, 2005 and June, 2007 and demonstrated that gefitinib combined with γ-ray SBRT is feasible and exhibits long-term and short-term efficacy.

## Patients and methods

### General

This study did not use randomization; instead, the opinions of the patients and their family members were solicited and the patients were subsequently assigned to the corresponding therapeutic group.

### Patient selection criteria

Criteria for enrollment in the study were as follows: adenocarcinoma of the lung confirmed by histology or cytology; operation and chemotherapy were rejected by the patients and/or their family members; the age of patients was ≥70 years and their performance status according to Zubrod-ECOG-WHO (ZPS) was 0–3; presence of at least one measurable lesion and a total number of lesions <3; normal function of the heart, liver and kidneys; no moderate or severe malignant hydrothorax; no typical interstitial pneumonia or pulmonary fibrosis. This study was approved by the ethics committee of Jiangsu University. Patients and/or family members signed the informed consent.

### Patient charcteristics

A total of 122 patients (54 males and 68 females) were included in the 3 groups (average age, 74.5 years; range, 70–83 years). All 122 patients had adenocarcinoma of the lung confirmed by histology or cytology. According to the American Joint Committee on Cancer TNM Staging System for lung cancer, 27 patients had stage II and 95 had stage III lung adenocarcinoma. The average tumor diameter was 5.6 cm (range, 2–10 cm). The ZPS performance status was 0–3 and the average score was 2.2. There was no statistical variance in age and clinical stage among the 3 groups (Table I).

### Therapeutic method

Group A included 35 patients treated with gefitinib combined with γ-ray SBRT, group B included 45 patients treated with γ-ray SBRT alone and group C included 42 patients treated with gefitinib alone. The patients received 250 mg of gefitinib per day, from the first day of the treatment until disease progression or discontinuation due to other causes. The patients were treated with γ-ray SBRT, initiated on the second day. The dose curve for this case group was 50–80%. The encircled dose was 4.0–6.5 Gy per fraction and the range of the total radiation dose was 36–48 Gy. The total number of treatments was 8–12, at a frequency of 5 times per week. Antiasthmatic, expectorant, anti-inflammatory and other symptomatic treatments were used as supportive therapy in order to manage symptoms such as cough, expectoration and shortness of breath.

### Assessments of therapeutic effects

The patients were telephonically followed up monthly and by a follow-up visit at the clinic once every 3 months. The objective treatment effectiveness was assessed in the 3 groups by evaluation of the double helical computed tomography (CT) performed at 2 months. Efficacy was evaluated using the Response Evaluation Criteria in Solid Tumors, version 1.1 (6). Patients were classified as exhibiting complete response (CR), partial response (PR), stable disease (SD) or progressive disease (PD). The response rate (RR) was calculated using the formula RR = CR + PR. The disease control rate (DCR) was calculated using the formula DCR = CR + PR + SD. Toxicity was evaluated according to the standards for adverse reactions (grades 1–4), as issued by the National Cancer Institute of the USA (7).

### Statistical analysis

The therapeutic effect was evaluated as the time period between treatment effectiveness and appearance of signs of disease progression. The OS rate was estimated for the time period between enrollment and death. Disease progression was evaluated from enrollment to the appearance of signs of disease progression. The objective therapeutic effect was calculated with the χ^2^ test and the survival time was assessed using the Kaplan-Meier method. All 122 patients enrolled in our study were analyzed.

## Results

### Objective therapeutic effects

**Figure 1.**
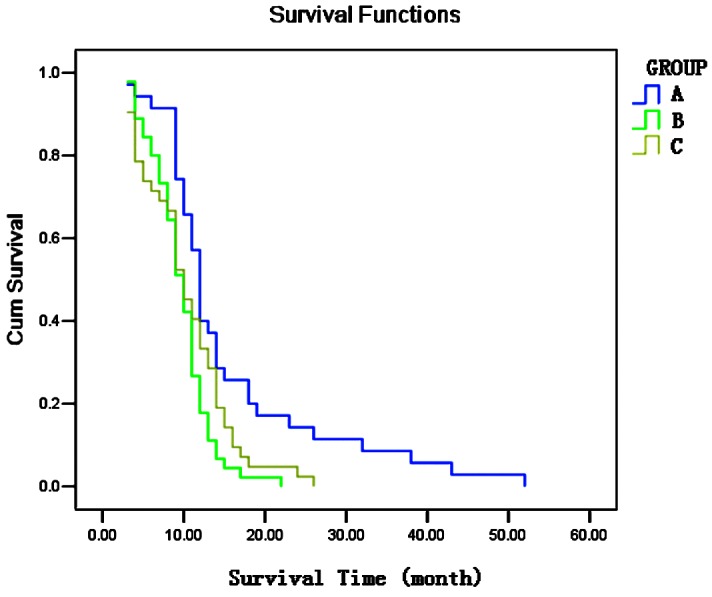
Kaplan-Meier estimates of overall patient survival from the time of of treatment initiation.

Table II shows the effects exerted by different therapeutic methods, as assessed by contrast-enhanced double helical CT at 2 months and expressed as progression-free survival (PFS), median overall survival (OS) and 1-year OS rate. The survival analysis is shown in Fig. 1. Group A exhibited a better short-term therapeutic effect (RR) compared to that of group C (P=0.014); however, there was no significant difference compared to group B (P=0.116). The PFS of group A was higher compared to that of groups B and C (7.8 vs. 5.9, P=0.018 and 7.8 vs. 5.1, P=0.013, respectively). The OS of group A was higher compared to that of groups B and C (15.5 vs. 9.6, P=0.002 and 15.5 vs. 10.3, P=0.017, respectively). The short-term therapeutic effect in group B was better compared to that in group C, although this finding was of no statistical significance (P=0.320). There were no significant differences in PFS and OS between groups B and C (5.9 vs. 5.1, P=0.329 and 9.6 vs. 10.3, P=0.633, respectively).

### Adverse effects

All 122 patients were included in the toxicity evaluation (Table III). Adverse effects experienced by patients were considered acceptable and they included skin rash and diarrhea (grades 1–3) that were manageable by symptomatic treatment.

## Discussion

Gefitinib is an aniline quinazoline compound that may be used to promote apoptosis, limit angiogenesis and restrict differentiated proliferation or migration of cancer cells via the potent inhibition of EGFR tyrosine kinase. It is an oral pharmaceutic and may be administered at home. Therefore, it is widely used as second- or third-line treatment in patients with advanced NSCLC, following failure of platinum-based therapy (7). Although a previous ISEL study (8) did not demonstrate a distinct effect on the prolongation of the patient life span, the subordinate analysis demonstrated a significant life span prolongation in patients of Asiatic origin. The median survival time of the gefitinib and placebo groups was 9.5 and 5.5 months, respectively. Wu *et al* (9) reported that the parameters resulting from gefitinib administration to Chinese patients with advanced NSCLC were as follows: CR, 4.3% (5/115); PR, 39.1% (45/115); NC, 27.0% (31/115); PD, 29.6% (34/115) and RR, 43.5% (50/115); the median time of symptomatic response was 8 days. These results demonstrated that gefitinib exerted satisfactory therapeutic and few adverse effects in Chinese patients with advanced NSCLC. Recently, Gao *et al* (10) reported their clinical observations from a study investigating gefitinib as a first-line treatment in 68 patients with advanced NSCLC and reached the conclusion that first-line gefitinib treatment is an effective and well-tolerated treatment regimen for advanced NSCLC. The complications of conventional radiotherapy are limiting to its application in senile lung cancer patients, due to their poor overall health status. With the development of stereotactic radiological technology, we may focus the delivery of the appropriate radiation dose to the diseased region, avoiding irradiation of surrounding normal tissues and organs. Thus, we may increase irradiation dose of the tumor while protecting the normal tissues. Therefore, the complications of radiotherapy are reduced, total treatment time is shortened and SBRT may be successfully used in senile lung cancer patients. To the best of our knowledge, SBRT is effective regarding locoregional tumor control; however, its therapeutic effect regarding tumor recurrence or metastasis is limited. Therefore, we attempted to combine gefitinib and SBRT in a treatment regimen that is locally and systemically effective for the senile NSCLC patients. Four previously conducted large (>4,000 patients) randomized phase III trials on chemotherapy with or without concomitant administration of an EGFR tyrosine kinase inhibitor in unselected patients with advanced-stage NSCLC, did not demonstrate any correlation between combination treatment and improvement in clinical outcome (11). However, a preclinical study conducted by Tanaka *et al* (12) demonstrated that gefitinib enhances the radioresponse of NSCLC cells by suppressing cell DNA repair capacity, thereby prolonging the presence of radiation-induced DNA double-strand breaks (DSBs). Their findings suggested that the combination of gefitinib and radio-therapy may be of value in clinical practice. A previous study by Zhuang *et al* (13) demonstrated that optimal radiosensitization was achieved when gefitinib was administered prior to irradiation. In our study, gefitinib treatment was initiated on the first day and radiation treatment was initiated on the second day, allowing for the position fixing of γ-ray SBRT on the first day. Therefore, gefitinib may have significantly enhanced the efficiency of radiotherapy in our study. Stinchcombe *et al* (14) reported that induction chemotherapy with carboplatin, irinotecan and paclitaxel, followed by high-dose three-dimensional conformal thoracic radiotherapy (74 Gy) with concurrent administration of carboplatin, paclitaxel and gefitinib, elicited a good response in unresectable stage IIIA and IIB NSCLC. However, the combination of gefitinib, radiotherapy and chemotherapy was associated with severe adverse effects, such as radiation esophagitis and cardiac toxicity. Therefore, we did not include chemotherapy in our combination treatment, since our clinical trial involved senile patients.

There was no reported patient mortality associated with treatment-related adverse effects. Furthermore, the adverse effects experienced by the patients were tolerable and treatable. The RR of group A (gefitinib combined with γ-ray SBRT), group B (γ-ray SBRT alone) and group C (gefitinib alone) was 68.6, 51.1 and 40.5%, respectively, when we assessed tumors with contrast-enhanced double helical CT at 2 months. The RR demonstrated by our study is higher compared to that observed in non-Asiatic patients, a finding that may be attributed to the fact that RR is positively correlated with the mutation rate of EGFR, which, to the best of our knowledge, is higher in Chinese compared to non-Asiatic patients (15,16). Group A exhibited better short-term therapeutic effects compared to group C (P=0.014) and had higher PFS and OS rates compared to groups B and C. It suggested that gefitinib combined with γ-ray SBRT exhibited better short- as well as long-term therapeutic effects when used as a first-line regimen for the treatment of senile patients with adenocarcinoma of the lung. Therefore, this trial demonstrated that the combination of micromolecular-targeted drug therapy and SBRT may be beneficial. Furthermore, group A (gefitinib combined with γ-ray SBRT) exhibited better short-term therapeutic effects compared to group B (γ-ray SBRT alone), although the difference was of no statistical significance (P=0.116). This finding may be attributed to our relatively small patient sample and further studies, including larger patient samples, are required. Previous studies demonstrated that the EGFR gene mutation status was associated with the efficacy of gefitinib in patients with advanced NSCLC (17,18). A recent NEJ 003 study suggested that first-line gefitinib treatment may be preferable to standard chemotherapy for advanced NSCLC patients aged ≥75 years harboring EGFR mutations (19). However, we did not screen all patients for EGFR and KRAS mutations at the initial enrollment in 2005. Therefore, we were not able to elucidate the relationship between gene mutation status and treatment efficacy for different treatment protocols.

We are currently observing the therapeutic effects of SBRT in patients not previously treated with gefitinib, using gefitinib only in the case of tumor recurrence following treatment with SBRT. At present, the therapeutic effects of a treatment protocol combining erlotinib and SBRT are under evaluation. Whether the combination of molecular-targeted therapy and radiotherapy is preferable to the current standard combination of chemo- and radiotherapy in non-senile patients with advanced NSCLC, requires further investigation.

In conclusion, gefitinib combined with γ-ray SBRT appears to be feasible and effective as the first-line treatment regimen for senile patients with adenocarcinoma of the lung, although further investigations are required.

## Figures and Tables

**Table I.** Patient characteristics.

Characteristic	Group A	Group B	Group C
Average age (years)	74.6	74.4	74.5
Male	16	20	18
Female	19	25	24
Average performance status (ZPS)	1.9	1.9	1.9
Average score	2.3	2.2	2.2
Average diameter of the lesions (cm)	5.2	5.7	5.7

**Table II.** Therapeutic effects in patients treated by different therapeutic methods.

Therapeutic method	CR n (%)	PR n (%)	SD n (%)	PD n (%)	RR n (%)	DCR n (%)	PFS (months)	OS (months)	1-year OS rate (%)
Group A	3 (8.6)	21 (60.0)	7 (20.0)	4 (11.4)	24 (68.6)	31 (88.6)	7.8	15.5	40.0
Group B	1 (2.2)	22 (48.9)	9 (20.0)	13 (28.9)	23 (51.1)	32 (71.1)	5.9	9.6	15.6
Group C	3 (7.1)	14 (33.3)	9 (21.4)	16 (38.1)	17 (40.5)	26 (61.9)	5.1	10.3	35.7

CR, complete response; PR, partial response; SD, stable disease; PD, progressive disease; RR, response rate; DCR, disease control rate; PFS, progression-free survival; OS, overall survival.

**Table III.** Adverse effects experienced by patients treated by different therapeutic methods.

Adverse effect	Group A, n (%)				Group B, n (%)				Group C, n (%)			
	0	1	2	3^a^	0	1	2	3^a^	0	1	2	3^a^
Leukopenia	25 (71)	7 (20)	3 (9)	0 (0)	36 (80)	5 (11)	4 (9)	0 (0)	40 (95)	2 (5)	0 (0)	0 (0)
Thrombocytopenia	32 (91)	3 (9)	0 (0)	0 (0)	43 (96)	2 (4)	0 (0)	0 (0)	41 (98)	1 (2)	0 (0)	0 (0)
Anemia	29 (83)	5 (14)	1 (3)	0 (0)	40 (89)	3 (7)	2 (4)	0 (0)	40 (95)	2 (5)	0 (0)	0 (0)
Rash	10 (29)	14 (40)	9 (26)	2 (6)	43 (96)	2 (4)	0 (0)	0 (0)	15 (36)	14 (33)	12 (29)	1 (2)
Diarrhea	19 (54)	7 (20)	6 (17)	3 (9)	43 (96)	2 (4)	0 (0)	0 (0)	24 (57)	9 (21)	8 (19)	1 (2)
Nausea/vomiting	26 (74)	5 (14)	3 (9)	1 (3)	41 (91)	4 (9)	0 (0)	0 (0)	37 (88)	3 (7)	2 (5)	0 (0)
Dyspnea	24 (69)	7 (20)	3 (9)	1 (3)	39 (87)	5 (11)	1 (2)	0 (0)	36 (86)	4 (10)	2 (5)	0 (0)

a Numbers 0–3, grade of severity of adverse effect.
